# Response rates in postal surveys of healthcare professionals between 1996 and 2005: An observational study

**DOI:** 10.1186/1472-6963-9-160

**Published:** 2009-09-14

**Authors:** Julia V Cook, Heather O Dickinson, Martin P Eccles

**Affiliations:** 1 Institute of Health and Society, Newcastle University, 21 Claremont Place, Newcastle upon Tyne, NE2 4AA, UK

## Abstract

**Background:**

Postal surveys are a frequently used method of data collection in health services research. Low response rates increase the potential for bias and threaten study validity. The objectives of this study were to estimate current response rates, to assess whether response rates are falling, to explore factors that might enhance response rates and to examine the potential for non-response bias in surveys mailed to healthcare professionals.

**Methods:**

A random sample of postal or electronic surveys of healthcare workers (1996-2005) was identified from Medline, Embase or Psycinfo databases or Biomed Central. Outcome measures were survey response rate and non response analysis. Multilevel, multivariable logistic regression examined the relationship between response rate and publication type, healthcare profession, country and number of survey participants, questionnaire length and use of reminders.

**Results:**

The analysis included 350 studies. Average response rate in doctors was 57.5% (95%CI: 55.2% to 59.8%) and significantly lower than the estimate for the prior 10 year period. Response rates were higher when reminders were sent (adjusted OR 1.3; 95%CI 1.1-1.6) but only half the studies did this. Response rates were also higher in studies with fewer than 1000 participants and in countries other than US, Canada, Australia and New Zealand. They were not significantly affected by publication type or healthcare profession (p > 0.05). Only 17% of studies attempted assessment of possible non-response bias.

**Conclusion:**

Response rates to postal surveys of healthcare professionals are low and probably declining, almost certainly leading to unknown levels of bias. To improve the informativeness of postal survey findings, researchers should routinely consider the use of reminders and assess potential for non-response bias.

## Background

Postal surveys are commonly used to gather information from healthcare professionals. It is important that studies using survey methodology minimise or at least recognise the influence of non-responders, as this can undermine study validity and thus generalisability to a wider population.

Health professionals, in particular doctors, are considered to be a problematic population from which to collect survey data [1]. Average response rates amongst doctors were reported to be 61% in studies published during the 10 year period 1986-1995 [2] and a comparable figure of 62% was reported for mail surveys published in US medical journals in 1991 [1] although only 50% of the included surveys were amongst health professionals. The study by Cummings et al [2], considered the influence of the number of survey participants suggesting that response rates were higher in surveys with fewer than 1000 participants. Asch et al [1], considered a wider set of explanatory variables (respondent characteristics: profession, age, gender; survey characteristics: reminders, anonymity, survey length, postage and use of financial incentives) and suggested that doctors had a lower response rate compared to non-doctors, and using written or telephone reminders was associated with a higher response.

However, there are concerns that response rates may have fallen recently due to increasing demands to participate in research activities [3,4]. Low response rates can result in bias, as non-responders may be systematically different from responders [5] and thus non-response analysis is important in interpreting survey results. Although it is impossible to know for sure whether non-response has introduced bias, several techniques are available for assessing the likelihood of this [6]. Whilst a response rate of 75% is considered an acceptable minimum standard [7], higher response rates are important to reduce the potential for bias due to non-response. Two large systematic reviews [8,9] of interventions to increase survey response rates (inclusive of both the general public, patients and healthcare professionals) identified factors that enhance response rates (monetary incentives, recorded delivery systems, shorter questionnaires, saliency of the survey topic, use of reminders and prenotification contact). Two smaller systematic reviews of randomised controlled trials that specifically focused on healthcare professionals [10,11] found the use of monetary incentives, reply paid envelopes, shorter questionnaires, recorded delivery and survey personalisation to increase survey response.

We have therefore updated the response rate analysis of Cummings et al [2] taking into account a range of potential factors known to influence response rate. The objectives of this study were to estimate response rates to postal questionnaires targeting healthcare professionals in studies published in the 10 year period 1996-2005, to assess whether response rates among doctors had fallen since the preceding 10 year period, to explore the influence of multiple factors associated with higher response rates and to determine the frequency of assessment of potential for non-response bias.

## Methods

### Database selection and search strategy

Studies with low response rates may be less likely to be published in journals with space limitations. We therefore compared surveys published in Biomed Central, which publishes only electronically and has no restriction on article length, with surveys indexed in "standard" bibliographic databases Medline, Embase and Psycinfo. These databases were selected to give comprehensive international coverage and to include both medical and psychosocial disciplines.

The search strategy is detailed below.

#1 survey$ or questionnaire$.tw.

#2 clinician$ or dentist$ or doctor$ or family practition$ or general practition$ or GP$ or FP$ or gyn?ecologist$ or hematologist$ or haematologist$ or internist$ or nurse$ or obstetrician$ or occupational therapist$ or OT$ or pediatrician$ or paediatrician$ or pharmacist$ or physician$ or physiotherapist$ or psychiatrist$ or psychologist$ or radiologist$ or surgeon$ or therapist$ or counse?lor$ or neurologist$ or optometrist$ or paramedic$ or social worker$ or health professional$ or primary care).tw.

#3 1 and 2

Inclusion criteria are described in Table 1. Studies using a postal or electronic survey methodology (published during 1996-2005) were identified by searching the databases mentioned above. References were downloaded, duplicates were removed and Biomed references were excluded from the standard database set.

**Table 1:** Inclusion criteria and data abstraction protocol

	**Inclusion criteria**	**Exclusion criteria**
**Surveys**	Self completed mail surveys including electronic surveys using fax, internet or email. Health care topic. Minimum 100 participants. Published in English. Reported response rate.	Telephone surveys, personal interviews and captive audience surveys.

**Participants**	Healthcare professionals including doctors, nurses, allied health professionals, such as pharmacists, dentists, occupational therapists, physiotherapists and radiographers.	Students, patients and general public.

	**Data Abstraction**	

**Final response rate**	Number of usable questionnaires (completed or partially completed questionnaires) returned after the final reminder, divided by the total number sent.	

**Number of survey participants**	Studies were grouped into <250, 250-499, 500-749, 750-999, 1000-2499, > = 2500 participants.	

**Publication Type**	Standard or electronic format.	

**Health professional**	Doctor, nurse, allied health or mixed populations.	

**Country of study population**	Studies were grouped into US, Canada, Australia/New Zealand, UK/Ireland, other European and other countries.	

**Questionnaire length**	Time required to complete questionnaire was used as a proxy for questionnaire length and estimated 3 pages or 8 questions per minute. Studies were grouped as <10 minutes, 10-19 minutes and > = 20 minutes.	

**Number of reminders**	Studies were grouped into no reminders, 1 reminder, 2 reminders and 3-5 reminders. Reminders could be postal, telephone or electronic.	

**Use of financial incentives**	Cash or prize draw incentive.	

**Survey Type**	Surveys were classified as electronic if they were distributed by solely electronic processes. Those using only postal distribution or a mixed postal and electronic design were classified as postal.	

### Sampling procedure and data abstraction

All references published in electronic media were screened for inclusion but references in the standard databases were sampled before screening. All screening was performed by JVC. We estimated that 272 studies were required to detect a change of 5% at significance p < 0.05, 80% power with a cluster size of 100 and an intracluster coefficient of 0.08 [12,13], assuming a baseline response rate of 61% [2]. We assumed that only 1 in 7 references would fulfil the inclusion criteria and therefore screened 2000 references, which were randomly selected using computer generated sequences of 200 random numbers per year.

Data abstraction parameters are set out in Table 1. Number of survey participants, type of healthcare professional, questionnaire length, use of reminders and financial incentives were selected as predictors of response rate based on data from studies of health professionals and the general population. Publication type was chosen in order to examine the potential for publication bias. Country of study population was included as country specific factors such as healthcare system and net remuneration could moderate the effects of financial incentives. Non-response analysis was deemed present if researchers compared demographic variables between respondents and non-respondents, demonstrated sample representativeness or contacted a sample of those who did not reply.

### Statistical methods

We examined the effects on response rate of publication type, healthcare profession, country of study population, length of questionnaire, number of reminders and number of study participants using multivariable, multilevel logistic regression that allowed for clustering of healthcare professionals within studies [14]. The primary outcome measure was whether or not a healthcare professional had responded to a questionnaire; these individual responses were combined in the response rate for each study.

Multilevel modelling was used because the likelihood of response by different healthcare professionals within the same study is likely to be more similar than that by healthcare professionals in different studies. The multilevel model gives more weight to larger studies. It assumes that the response rate in a study with specific characteristics is sampled from a normal distribution and estimates the mean and variance of this distribution.

Initially, multilevel univariate logistic regression was performed, considering in turn each covariate, categorised as in Table 2. One third of the studies (108) did not provide information about length, so unreported length was considered as a separate category in the analysis. Covariates were selected for the multivariable model using a forwards stepwise procedure and the likelihood ratio test statistic (LRTS) to compare nested models. All variables significant in the multivariable analysis were tested for removal with a backwards step at each stage. To lessen the probability of chance findings due to multiple hypothesis testing, the p-value p < 0.01 was deemed to be significant for entry and removal of covariates. Categories within variables were collapsed if this made no significant difference. The final multivariable model excluded studies with missing values on the included covariates and was therefore based on 339 (96%) of the included studies. We checked for significant interactions between covariates in the final model. Odds ratios (OR) and their 95% confidence intervals (CI) are reported. From the final model, we estimated the overall mean response rate and its 95% confidence interval (which contains the actual mean with a probability of 0.95).

**Table 2:** Descriptive statistics and odds ratios (ORs) from univariate multilevel models

**Categories**	**Number of studies**		**Number of participants**		**Response rate (%)**		**Odds ratio**		
	n	(%)	n	(%)	Med.	IQR*	OR	95%CI**	p†

**Health professional**									0.18

Doctor	236	(67)	219,859	(60)	59	44 to 72	1.00	-	

Nurse	36	(10)	64,377	(18)	50	37 to 71	1.05	0.71 to 1.55	

Allied	57	(16)	59,872	(16)	57	35 to 68	0.81	0.63 to 1.05	

Mixed	21	(6)	21,382	(6)	62	45 to 74	0.77	0.57 to 1.05	

**Country**									< 0.0001

US	130	(37)	179,589	(49)	51	38 to 65	1.00	-	

Canada	34	(10)	36,976	(10)	53	38 to 67	1.13	0.82 to 1.55	

Australia/New Zealand	20	(6)	14,736	(4)	53	38 to 68	1.13	0.76 to 1.68	

UK/Ireland	82	(23)	51,790	(14)	62	52 to 76	1.69	1.34 to 2.14	

Other European countries	48	(14)	59,235	(16)	63	59 to 73	1.72	1.30 to 2.28	

Other countries	36	(10)	23,164	(6)	61	43 to 76	1.63	1.19 to 2.22	

**Length (minutes)**									0.0005

<10	123	(35)	100,794	(28)	55	40 to 70	1.00	-	

10-19	77	(22)	103,547	(28)	51	38 to 66	0.92	0.72 to 1.18	

> = 20	42	(12)	62,465	(17)	52	40 to 67	0.94	0.70 to 1.27	

No information	108	(31)	98,684	(27)	64	53 to 76	1.47	1.17 to 1.83	

**Number of reminders**									0.08

0	176	(50)	176,886	(48)	53	38 to 70	1.00	-	

1	79	(23)	75,253	(21)	60	49 to 68	1.25	0.99 to 1.57	

2	47	(13)	41,995	(11)	61	51 to 74	1.28	0.97 to 1.70	

3-5	37	(11)	56,207	(15)	62	50 to 72	1.35	0.99 to 1.83	

No information	11	(3)	15,149	(4)	52	38 to 72	-	-	

**Number of survey participants**									< 0.0001

> = 2,500	34	(10)	177,159	(48)	43	26 to 62	1.00	-	

1000-2499	62	(18)	87,362	(24)	46	38 to 59	1.16	0.83 to 1.61	

750-999	22	(6)	19,004	(5)	51	38 to 60	1.32	0.86 to 2.03	

500-749	61	(17)	36,674	(10)	53	40 to 63	1.46	1.04 to 2.04	

250-499	86	(25)	30,546	(8)	65	47 to 75	2.19	1.59 to 3.01	

<250	85	(24)	14,745	(4)	66	58 to 75	2.66	1.93 to 3.66	

**Publication type**									0.51

Electronic	75	(21)	75,409	(21)	60	44 to 71	1.00	-	

Standard	275	(79)	290,081	(79)	58	44 to 71	0.93	0.74 to 1.16	

**All studies**	350	(100)	365,490	(100)	59	42 to 71			

* IQR = interquartile range

** 95%CI = 95% confidence interval

† p value from likelihood ratio test statistic comparing models with and without the listed categories of the specified factor

Odds ratios above 1.0 indicate a higher response rate in that category than in the reference group.

Cook's distance [15] was used to identify studies with undue influence.

## Results

### Description of studies

From 123,538 references downloaded from the standard databases, 2,000 were randomly sampled; 277 fulfilled the inclusion criteria. Of the 494 references from Biomed Central, 75 fulfilled the inclusion criteria. The median number of participants in these 352 studies was 275 (interquartile range: 150 to 498).

Two very large studies, originating in Canada [16] and the US [17] with 61,751 and 51,672 participants respectively were excluded from the regression analysis because preliminary analysis indicated that they had undue influence on the results. The remaining 350 studies surveyed 365,490 healthcare professionals.

Two thirds of the included studies were in doctors, nearly a third did not report questionnaire length and a third required less than 10 minutes to complete (Table 2). Nearly all studies reported the number of reminders used: half did not use reminders. Thirty-five different countries were represented. Thirty seven percent of studies were based in the US and 23% were in the UK/Ireland. Other European countries accounted for 14% of studies; the only other countries with more than 10 studies were Canada (10%) and Australia/New Zealand (6%).

Various characteristics of studies were significantly correlated. On average: longer studies were larger and had more reminders (Spearman's rank correlation (ρ) = 0.22, 0.20; p = 0.0006, 0.002 respectively); US-based studies were longer and larger (ρ = 0.17, 0.18; p = 0.01, 0.009 respectively); studies in Europe were less likely to report the length of the study (ρ = 0.13, p = 0.02); studies of doctors were shorter (ρ = 0.21, p = 0.001). Among studies published electronically, three-quarters were published between 2004 and 2005 and all except two were conducted in Europe. Too few studies (3%) reported the use of financial incentives or a solely electronic design (3%) to allow further analysis of these factors.

### Response rates

The simple mean of response rates (giving equal weight to studies of all sizes) was 56% (95%CI: 54.4% to 58.3%). The median response rate was 59% (interquartile range 42.2% - 70.8%). Only 56 studies (16%) reported response rates over 75%.

Univariate logistic regression (Table 2) showed no significant difference in response rates between surveys of different types of healthcare professionals (p = 0.18), or between surveys published in standard and electronic format (p = 0.51). Survey response rates tended to be higher in studies with more reminders and those of unknown length and lower in the US, Canada, Australia and New Zealand and in larger studies. Adjacent categories were collapsed (Table 3) if their response rates were not significantly different. Multivariable logistic regression (Table 3) confirmed the associations found in univariate analysis, but with a less marked relationship between response rate and country, as the lower response rate in studies in the US and Canada was partly explained by the larger size of these studies. We found no statistically significant interactions between covariates. After allowing for these associations, substantial unexplained variation in response rates remained. Some variation is to be expected because of the effect of sampling within studies. However, the intra-cluster correlation coefficient (ICC = 0.16) indicated that most of the unexplained variation (84%) was between studies. In sensitivity analyses, each of the very large, excluded studies [16,17] was included in turn in the final multivariable model; this yielded similar estimates of the effect of the included factors, but with more variation between studies.

**Table 3:** Odds ratios (ORs) from final multivariable multilevel model

**Categories**	**Number of:**		**Odds ratio**		
	
	**Studies**	**Participants**	**OR**	95%Cl**	**p†**
**Country**					< 0.0001

US, Canada, Australia, New Zealand	178	222,016	1.00	-	

All other countries	161	128,325	1.34	1.13 to 1.60	

**Number of reminders**					0.0009

None	176	176,886	1.00		

One or more	163	173,455	1.33	1.13 to 1.57	

**Number of survey participants**					< 0.0001

> = 1,000	92	252,428	1.00	-	

500-999	80	53,345	1.30	1.03 to 1.63	

<500	167	44,568	1.97	1.61 to 2.41	

**Length**					0.0008

Length reported	234	255,802	1.00	-	

Length not reported	105	94,539	1.37	1.14 to 1.64	

** 95%Cl = 95% confidence interval

† p value from likelihood ratio test statistic comparing models with and without the listed categories of the specified factor

Eleven studies were excluded because they did not report the number of reminders.

Odds ratios above 1.0 indicate a higher response rate in that category than in the reference group.

### Comparison with previous studies

As Cummings' study was restricted to doctors, we compared Cummings' results with our results for all studies in doctors (including the largest study [16]). The simple mean response rate to questionnaires mailed to doctors (giving equal weight to studies of all sizes) in our study (1996-2005) was 57.5% (95%CI: 55.2% to 59.8%), based on 237 surveys. For surveys of doctors published between 1986 and 1995, Cummings et al [2] reported that the simple mean response rate for doctors was 61.2%, based on 257 surveys, but did not report confidence intervals on this estimate. If we assume that the variation between response rates in Cummings' study was similar to that for surveys of doctors in our study, then the 95% confidence interval on Cummings' response rate would be 59.0% to 63.4%. A two-sided t-test indicated that our estimate and Cummings' estimate are significantly different (p = 0.02), confirming a small decrease in the mean response between 1986-1995 and 1996-2005.

### Frequency of assessing potential for non-response bias

Fifty-eight of 350 studies (17%) reported some form of non-response analysis. Thirty-three compared socio-demographic characteristics of respondents and non-respondents. Frequently reported characteristics were age and gender, but also reported were: workplace location (hospital, GP, community), setting (urban, rural), size (single handed, multiple partners) and individual characteristics e.g. speciality, affiliation with professional bodies or university, years since graduation. Eleven studies assessed sample representativeness by comparing respondents' socio-demographic characteristics with those of a national database or large national survey. Four studies conducted telephone/personal interviews in subsets of non-responders. Three studies examined differences between early and late responders. Finally, four studies used multiple strategies, and three studies claimed to analyse non-response but did not report how.

## Discussion

This study showed that the response rate to postal surveys in studies of healthcare professionals published between 1996 and 2005 was low: 56% (95%CI 54% to 58%). Response rates showed wide variations, tending to be lower in larger studies and in studies in the US, Canada, Australia and New Zealand and higher in surveys that sent reminders, but only half the studies sent reminders. Few studies reported an analysis of non-responders.

For surveys of doctors, we found a small but statistically significant decrease in response rates compared to the previous 10 years [2], from an average of 61.2% to 57.5%. Any differences in response rate between our study and Cummings' could be influenced by differences in the characteristics of studies e.g. country, number of survey participants, and number of reminders published in the respective 10-year periods. However, our study had a higher percentage of small surveys in doctors (with less than a thousand participants) than that of Cummings - 73% (173/237) compared to 67% (173/257) - and, since smaller surveys tend to have higher response rates, we would have expected our study to find a higher average response rate than Cummings' if other factors were similar.

### Strengths and weakness of this work

We updated Cummings' study [2] of response rates to questionnaires mailed to doctors in the ten years from 1986-1995 by considering the following 10-year period. We included healthcare professionals other than doctors, but found no significant differences in response rates between professional groups. Although we selected surveys from the major health-related electronic bibliographic databases - Medline, Embase and Psycinfo - we did not include a database that was specifically focused on nursing e.g. Cinahl, largely to ensure comparability with Cummings' study [2]. Unlike Cummings, we modelled the association between study characteristics and response rate while allowing for a propensity towards similar responses by healthcare professionals within the same study. Despite examining a core set of recognised variables, much of the variation between studies could not be explained by factors that we considered and further exploration would require extensive contact with authors. In addition, due to poor reporting in primary studies, we were unable to examine the influence of factors, such as financial incentives, mail delivery systems and importance of survey topic, that are known to influence response rates in the general population.

### Factors that influence response rates

Only 16% of studies achieved the response rate of 75% or over which is often regarded as the acceptable minimum [7]. Although our study confirmed the general consensus that reminders are an effective strategy to augment response rates [8,9,18], it was surprising that half the studies did not use any reminders. Even in the most favourable circumstances - studies outside the US, Canada, Australia and New Zealand, with reminders and less than 500 participants - the average response rate was only 65.5%.

It is unclear why larger studies and studies conducted in the US, Canada, Australia and New Zealand tended to have poorer response rates. The determinants of high response rates may be different in these countries and other countries; our study may not have captured the factors determining such differences. Smaller studies may have yielded higher response rates as they may focus more closely on issues salient to participants. The higher response rate in surveys that did not report length may be because surveys in Europe were less likely to report length but tended to have higher response rates.

### Analysis of non-response

Despite low response rates, only 17% of studies attempted any sort of assessment of the potential for non-response bias - a figure virtually identical to that seen by Cummings et al. [2]. Non-response analysis may be difficult and expensive: it requires assessing whether non-responders would have answered the questionnaire differently from responders, in some systematic way. If we assume that the propensity to non-response depends on the known characteristics of participants - e.g. basic demographic characteristics such as age, gender, profession - we can infer how non-responders would have answered, based on how responders with those characteristics did so. However, obtaining information even about such basic characteristics of non-responders may be problematic. If non-response depends on unknown characteristics of participants, then it is much more difficult to say how non-responders might have answered the questionnaire. In particular, if the reason for non-response is associated with the outcome of interest, bias will be inevitable. If this is suspected, it may be helpful to perform sensitivity analyses to explore how different assumptions about the determinants of non-response influence the conclusions [6]. This can be done either by explicitly modelling the probability of non-response, ideally using prior information from experts, or by "multiple imputation": replacing missing data by values randomly selected from a plausible distribution that reflects the postulated bias. However, all these methods of allowing for non-response are essentially informed guesswork and cannot compensate for the definitive knowledge provided by high response rates.

## Conclusion

Response rates to postal surveys of healthcare professionals are low and appear to be declining. Reminders are known to improve response rates yet only half of the studies used reminders. Although an assessment of the potential for non-response bias is crucial to the interpretation of study findings, such non-response analysis is seldom conducted. Journal readers should be very cautious about the results of any survey that does not report its response rates and discuss the possibility of non-response bias. If the scientific community wish to have reliable and valid information from postal surveys of healthcare professionals then a number of steps are required. Researchers should routinely conduct (and if necessary improve the methods of) non-response analysis. Research funders should allocate the additional resources required to conduct non-response analysis. Finally, journal editors should consider not publishing studies that have low response rates especially if the studies make no attempt to understand the implications of this.

## Competing interests

The authors declare that they have no competing interests.

## Authors' contributions

The guarantor of this paper is MPE. MPE had the original idea for the paper, HOD carried out the statistical analysis and JVC conducted the study. The manuscript was drafted by JVC and critically revised for intellectual content by HOD and MPE. All authors approved the final version of the manuscript.

## Pre-publication history

The pre-publication history for this paper can be accessed here:


